# Review of the Perspectives and Study of Thermo-Responsive Polymer Gels and Applications in Oil-Based Drilling Fluids

**DOI:** 10.3390/gels9120969

**Published:** 2023-12-11

**Authors:** Jintang Wang, Lei Liu, Siyang Zhang, Bo Liao, Ke Zhao, Yiyao Li, Jiaqi Xu, Longqiao Chen

**Affiliations:** 1 State Key Laboratory of Unconventional Oil & Gas Development, China University of Petroleum (East China), Ministry of Education, Qingdao 266580, China; s22020066@s.upc.edu.cn (L.L.); s22020048@s.upc.edu.cn (K.Z.); z23020088@s.upc.edu.cn (Y.L.); s23020130@s.upc.edu.cn (J.X.); 2 School of Petroleum Engineering, China University of Petroleum (East China), Qingdao 266580, China; 2002010631@s.upc.edu.cn; 3 CNPC Offshore Engineering Company Limited, Beijing 100028, China; chenlq6.cpoe@cnpc.com.cn

**Keywords:** thermo-responsive polymer gels, phase transition temperature, oil-based drilling fluids, oil and gas exploration

## Abstract

Thermoresponsive polymer gels are a type of intelligent material that can react to changes in temperature. These materials possess excellent innovative properties and find use in various fields. This paper systematically analyzes the methods for testing and regulating phase transition temperatures of thermo-responsive polymer gels based on their response mechanism. The report thoroughly introduces the latest research on thermo-responsive polymer gels in oil and gas extraction, discussing their advantages and challenges across various environments. Additionally, it elucidates how the application limitations of high-temperature and high-salt conditions can be resolved through process optimization and material innovation, ultimately broadening the scope of application of thermo-responsive polymer gels in oil and gas extraction. The article discusses the technological development and potential applications of thermo-responsive polymer gels in oil-based drilling fluids. This analysis aims to offer researchers in the oil and gas industry detailed insights into future possibilities for thermo-responsive polymer gels and to provide helpful guidance for their practical use in oil-based drilling fluids.

## 1. Introduction

Environmental stimulus-responsive polymers are a type of intelligent materials that can receive and respond to stimuli from the external environment (pH, temperature, light, electric field, magnetic field, etc.), which has become a hot research direction in the field of materials science in recent years, attracting more and more attention from researchers [1,2,3,4]. Temperature is one of the most straightforward stimulus factors to control the stimulus conditions of polymers. Among the different stimulus-responsive polymers, the materials that can respond to external temperature changes are called thermo-responsive polymers. Thermo-responsive polymers react rapidly and intensely to temperature, making research on them the most extensive. The diversity of thermo-responsive polymers is reflected in their ability to assume various forms ranging from gels and nanoparticles to films and micelles [5,6,7].

Thermo-responsive polymer gels, recognized as a unique category of intelligent materials, have attracted widespread interest owing to their capacity to sense and respond to environmental temperature variations [8,9]. Hydrophilic and hydrophobic chains, as well as intermolecular hydrogen bonds, are present in thermo-responsive polymer gels. Temperature changes directly impact the hydrophilic and hydrophobic interactions between the polymer chains and the aqueous solvent, leading to phase transition phenomena [10]. Characterized by a critical phase transition temperature, they exhibit pronounced changes in chemical properties or physical configurations, such as the dramatic swelling or deswelling of polymer gels or the phase transition from clarity to turbidity in polymer solutions [11,12]. Their structural states can be meticulously tailored by adding surfactants, ionic liquids, and salts or by manipulating the polarity and pH of the solvent [13,14,15]. In recent decades, these materials have secured a broad range of applications across multiple disciplines, notably biomedicine, consumer products, sensing technology, and environmental protection, propelled by their superior performance and controllable responsive traits.

In biomedicine, thermo-responsive polymer gels serve as carriers for drug loading and storage, utilized for targeted therapy and precise control over drug release, as well as scaffolding materials in tissue engineering to foster cell growth and tissue regeneration [16,17,18]. Incorporating thermo-responsive polymer gels into textile materials allows clothing to sense ambient temperatures. By adjusting the breathability and moisture permeability of the fabric at varying temperatures, wearers can experience enhanced comfort [19,20]. Thermo-responsive polymer gels typically feature reactive functional groups such as carboxyl, hydroxyl, amino, amide, and sulfonic acid. These groups engage in strong coordination interactions with heavy metal ions, enabling the removal of heavy metal ions produced in industrial processes through ion exchange or chelation, thereby facilitating wastewater treatment [21,22,23].

As the global exploitation of oil and gas resources intensifies, conventional extraction methods are increasingly challenged. This is particularly evident in high-temperature, high-pressure environments, such as deep-water drilling and unconventional hydrocarbon development, where efficient, safe, and environmentally friendly extraction methods are becoming a focal point of the industry [24,25,26]. In this context, thermo-responsive polymer gels, as environmentally sensitive innovative materials, demonstrate significant advantages and potential in oil and gas extraction, especially in oil-based drilling fluids applications. Throughout the various stages of oil and gas extraction, such as drilling, oil recovery, and post-production processing, thermo-responsive polymers are an innovative and intelligent material that significantly enhances operational efficiency while overcoming technical challenges. During drilling, these polymers can adjust to the high temperatures and pressures found in deep-well drilling, resulting in enhanced drilling efficiency and safety by optimizing the rheological properties of drilling fluids through their unique thermo-responsive properties. In the oil recovery phase, the thermo-responsive polymers can improve oil-water separation by precisely regulating fluid behavior in the reservoir, resulting in increased healthy production and recovery efficiency. During post-production treatment, thermo-responsive polymers demonstrate substantial benefits in treating produced fluids, optimizing the separation process, and reducing environmental impact. The employment of these polymers in oil and gas extraction is increasing due to technological advancements and a deeper comprehension of the oil and gas field.

This paper provides a comprehensive introduction to thermo-responsive polymers from various perspectives. It summarizes the existing research on thermo-responsive polymers in three aspects: drilling, oil recovery, and oil–water separation in oil and gas extraction. Additionally, considering the performance characteristics of thermo-responsive treating agents, this paper offers an outlook on the prospects for thermo-responsive polymers in oil and gas extraction, emphasizing their application in oil-based drilling fluids.

## 2. The Classification of Thermo-Responsive Polymers

### 2.1. The Mechanism of Thermo-Responsive Polymer Phase Transition 

**Figure 1:**
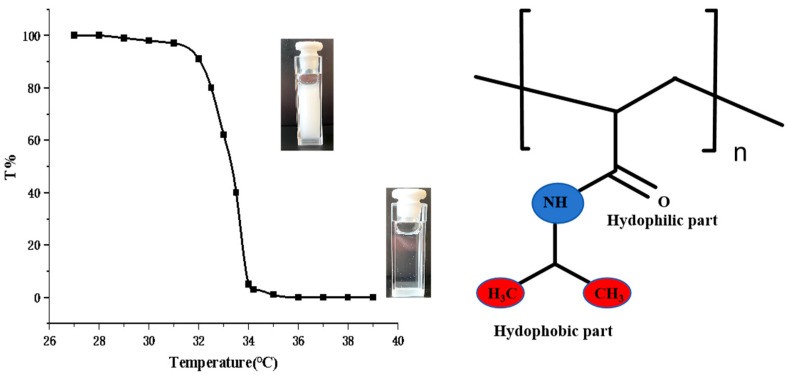
The structure of PNIPAM and its thermo-responsive phenomena.

The widely accepted view of the phase transition mechanism of thermo-responsive polymers posits that these polymers contain both hydrophilic and hydrophobic groups. In aqueous solutions, interactions between these groups and water molecules drastically alter the hydrophilic/hydrophobic balance of the polymer with temperature changes, causing the polymer solution to transition from a straightforward, uniform phase to a turbid solid-liquid phase. The temperature at which this transition occurs is known as the polymer’s phase transition temperature [27,28]. A typical thermo-responsive polymer, such as Poly (N-isopropyl acrylamide) (PNIPAM), contains hydrophilic amide groups (-CO-NH-) and hydrophobic isopropyl groups (-CH(CH_3_)_2_). As shown in Figure 1, when the temperature is below LCST, amide groups form hydrogen bonds with water molecules, causing polymer expansion and dissolution in water for uniform phases. Upon exceeding the LCST, the hydrophobic group’s action surpasses the hydrogen bond between the amide group and the water molecule, resulting in polymer contraction, aggregation, and phase separation [29].

Furthermore, according to the Flory–Huggins theory, a polymer solution’s change in free energy (ΔG_m_) can elucidate the phase transition mechanism of thermo-responsive polymers [30]. When the temperature is below the LCST (lower critical solution temperature) or above the UCST (upper critical solution temperature), the ΔG_m_ of the system is negative, and the polymer is in a dissolved state. Conversely, when the temperature exceeds the LCST or falls below the UCST, the system’s free energy becomes positive, causing the polymer to precipitate.

**Figure 2:**
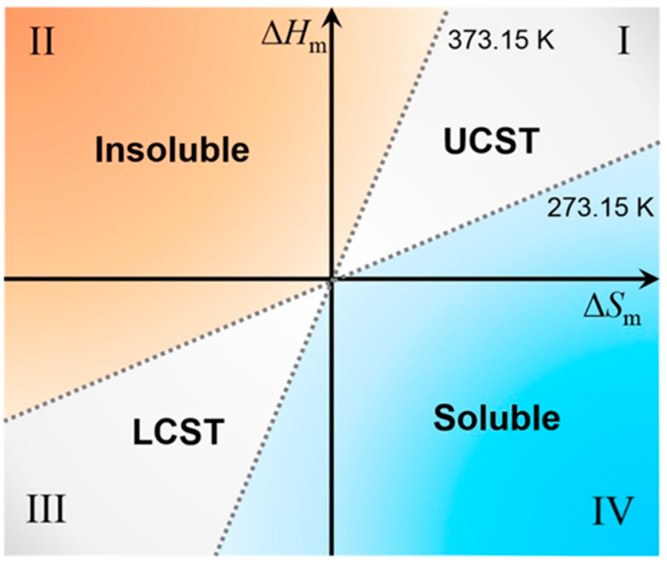
A thermodynamic map showing the solubility and solution properties of polymers in water [31].

Researchers created a coordinate system at a right angle based on enthalpy change (ΔH_m_) and entropy change (ΔS_m_) to plot the thermodynamic map of thermo-responsive polymers in aqueous solution, the tangent ∂ΔH_m_/∂ΔS_m_ of a line connecting the origin to a particular point in the ΔH_m_–ΔS_m_ quadrant is equal to the critical temperature (either UCST or LCST) for a given polymer system. Therefore, the critical temperature (T_c_) can be represented as T_c_= ΔH_m_/ΔS_m_ (Figure 2).

Thermo-responsive polymers can be classified into two categories based on their response to temperature changes: LCST and UCST [32]. LCST thermo-responsive polymers become insoluble in the solvent and exhibit immiscible phases when the temperature exceeds the phase transition temperature. Conversely, the polymer remains miscible with the solvent below this temperature. On the other hand, UCST thermo-responsive polymers exhibit characteristics that are the complete opposite of LCST polymers [33,34].

### 2.2. Thermo-Responsive Polymers with Lower Critical Solution Temperature

**Figure 3:**
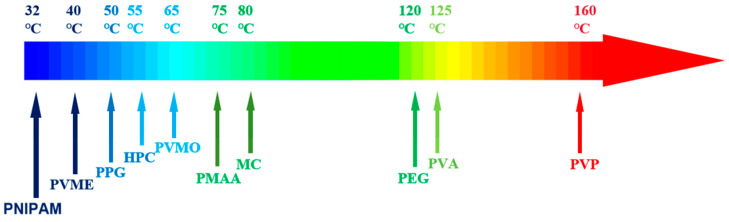
Common LCST thermo-responsive polymers.

The thermo-responsive polymers with LCST are numerous, as shown in Figure 3 and they are primarily categorized based on their chemical structures into Poly (Acrylamides), Poly (Ethers), Poly (vinyl amides), Poly (Methacrylates), and Poly (Oxazolines), among others [15,35]. 

### 2.3. Thermo-Responsive Polymers with Upper Critical Solution Temperature

Although UCST thermo-responsive polymers have a shorter application history than LCST, their notable performance characteristics, such as a broad UCST controllable range and low-temperature recovery properties, have made them a focal point for current research. UCST thermo-responsive polymers are generally divided into the following categories: Poly (Acrylamide) and its derivatives [36], Poly (N-Acryloylglycinamide) and its products [37,38,39], Poly (Urea) and its results [40], and Poly (Sulfobetaine) and its derivatives [41,42].

## 3. The Test Methods of the Phase Transition and the Control of Transition Temperature with a Thermo-Responsive Polymer 

To better understand the phase transition process of thermos-sensitive polymers, this section will focus on the determination of the phase transition temperature of thermos-sensitive polymers and its control methods, discussing how to accurately test and efficiently control the phase transition point of thermos-sensitive polymers, as well as the application of these techniques in practice.

### 3.1. Phase Transition Temperature Testing Methods

Amphiphilic thermo-responsive polymers change hydrophilicity and hydrophobicity due to variations in hydration action and weak intermolecular interactions around their phase transition temperatures. This leads to the polymer chains transitioning between dispersed and aggregated states, inducing significant changes in the system’s physical properties, such as optical transmittance, viscosity, and thermal effects. The polymer’s transition temperature can be determined by tracking the trend of a specific physical property of the solution.

#### 3.1.1. Differential Scanning Calorimetry

Differential scanning calorimetry (DSC) is used to detect the thermal effects of a polymer solution during a continuous temperature change. When the temperature of the polymer solution is near the phase transition point, the thermo-responsive polymer will undergo phase separation. The DSC captures the enthalpy change produced during the phase transition of the thermo-responsive polymer, and the temperature corresponding to the endothermic/exothermic peak indicates the phase transition temperature of the thermo-responsive polymer [43,44]. Liu et al. [45] determined the LCST of a hydrogel under different pH conditions by observing the DSC curves of swollen hydrogel samples, ultimately concluding that the thermo-responsiveness of the IPN-AAc hydrogel depends on the environmental pH value. Grinberg et al. [46] utilized high-sensitivity DSC to study the effect of different concentrations of β-glycerophosphate (GP) on the phase separation temperature of chitosan in aqueous solutions. Within the GP concentration range of 0.02−0.6 M, the overall change in the transition temperature of chitosan reached over 60 °C. Additionally, by fitting the DSC data, they estimated the standard free energy of β-glycerophosphate binding (Δbg_int_ = −6 ± 1 kJ/mol) and the cooperativity parameter (σ* = 1.78 ± 0.71).

DSC accurately determines the phase transition temperature of responsive polymers. However, the instrument’s high cost restricts its application in routine solution studies of these polymers.

#### 3.1.2. Swelling Degree Method

Thermo-responsive polymers display pronounced swelling or deswelling behavior around their critical dissolution temperatures (LCST or UCST). To assess this, samples of the thermo-responsive polymer are immersed in a solvent, with the temperature of the solution incrementally adjusted. Each specific temperature point is maintained for a determined duration. At every thermosensitive point, the swollen polymer’s mass or volume change is quantified, and the swelling data at various temperatures is documented. The point at which a sharp change in the degree of swelling occurs is designated as the phase transition temperature of the polymer [47]. 

PAN et al. [48] immersed dry samples of N-isopropyl acrylamide copolymer hydrogels in thermostatically controlled distilled water for a set duration, subsequently removing excess water from the gel surface with moist filter paper and then weighing the hydrogel. The swelling ratio is calculated using the formula Rs = (mt − md)/md, where t is the weight of the hydrogel after soaking for time t, and MD is the initial weight of the dry hydrogel. Given that the swelling kinetics are significantly influenced by factors such as the shape, size, crosslinking degree, internal pore structure, and pendant chains of the gel, these factors can dramatically affect the outcomes, making this method infrequently used for characterizing the phase separation temperature of polymers.

#### 3.1.3. Dynamic Light Scattering

Using UCST thermo-responsive polymers as an example, the polymer chains transition from an extended conformation to a more coiled state, leading to phase separation as the temperature of the polymer solution gradually decreases to the UCST. To monitor the changes in the molecular chains’ shape and size within the system before and after the phase transition, dynamic light scattering (DLS) can be utilized. The temperature range over which a sudden change in the molecular chains’ morphology is observed and is used to determine the phase transition temperature.

For thermo-responsive polymer gels, DLS can be used to measure the hydrodynamic size and distribution of particles within the system, with the temperature range of the abrupt change in particle size being indicative of the phase transition temperature [49]. Ribeiro et al. [50] utilized DLS to analyze the sol–gel transition of diluted solutions (0.01% *w*/*v*) of PNVCL, NC-5%-80, and NC-5%-330 within the temperature range of 33.0 to 34.6 °C, demonstrating that the sphericity within the nanocomposites begins to increase below the phase transition temperature, leading to a decrease in optical transmittance and the diffusive reflectance profile.

Generally, DLS instruments are user-friendly and do not necessitate a complex sample preparation process. Additionally, the equipment can provide thermo-responsive polymer particle size data quickly. Nevertheless, this method’s sample concentration requirements are more demanding, and both excessively high and low sample concentrations may impair the accuracy of measurements.

#### 3.1.4. Fluorescence Spectroscopy

After phase separation, thermo-responsive polymers form hydrophobic aggregates. By employing fluorescent probes or labels that are sensitive to the environment or based on the principle of aggregation-induced emission, the phase transition temperature of the polymers can be determined by the variation patterns of fluorescence intensity or peak ratios [51].

**Figure 4:**
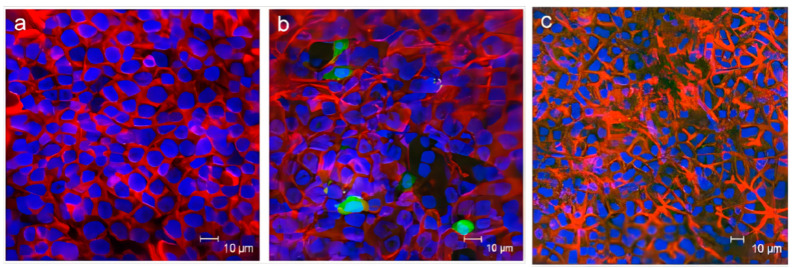
A novel thermo-responsive hydrogel for intranasal drug delivery: (**a**) CLSM images of rat nasal epithelia following administration of FITC-insulin solution in PBS, (**b**) FITC-insulin loaded HTCC–PEG–GP system, and (**c**) FITC-insulin solution in HTCC solution. [52].

Wu et al. [52] developed a novel thermo-responsive hydrogel for intranasal drug delivery, as shown in Figure 4. Using confocal laser scanning microscopy, they demonstrated that this formulation could enhance the absorption of insulin labeled with fluorescein isothiocyanate (FITC) within the nasal cavity of rats. The results indicated that this formulation could be employed as an intranasal drug delivery system to improve the absorption of hydrophilic macromolecular drugs.

Fluorescence spectroscopy methods offer high sensitivity and short response time and allow in situ measurements, making them valuable for monitoring phase transitions in thermo-responsive polymers. However, fluorophore selection requires caution, as its sensitivity to multifactorial conditions can alter fluorescence intensity, absorption of the fluorescence spectrum, and fluorescence lifetime parameters.

#### 3.1.5. Turbidity Method

Thermo-responsive polymers in solutions with LCST characteristics are clear and transparent at low temperatures. As the temperature rises, these polymers’ molecular chains begin to coil when reaching the LCST, causing the solution to turn from clear to turbid. However, the polymer solution becomes clear again when the temperature decreases below the LCST. In contrast, UCST thermo-responsive polymers behave oppositely. A simple and standard method to determine the phase separation temperature of these polymers is to measure the changes in light transmission (turbidity) before and after phase separation using UV-visible spectrophotometry [53]. Huang et al. [54] prepared a thermo-responsive hydrogel for drug delivery using chitosan (C) and β-glycerophosphate (β-GP). They determined that the prepared C/GP hydrogel underwent a rapid sol–gel transition within 5 min when placed in a 37 °C environment, as measured by turbidity methods.

The turbidity method is a standard and relatively convenient method for testing the phase transition temperature of thermoresponsive polymers. Nevertheless, the outcomes obtained only represent the phase transition phenomenon and are inadequate to disclose the mechanism.

#### 3.1.6. Viscosity Measurement Method

Near the phase transition temperature, thermoresponsive polymer molecular chains undergo coiling, aggregation, or stretching. When the polymer chains coil and aggregate, the solution’s viscosity increases; conversely, the viscosity decreases when the polymer chains uncoil. Based on this principle, the phase transition temperature of thermoresponsive polymers can be determined by tracking and measuring changes in the polymer solution viscosity [55].

Gan et al. [56] used potassium persulfate as an initiator to induce free radical polymerization between polyethylene glycol (PEG) macromonomers and chitosan chains, resulting in a temperature-responsive chitosan-polyethylene glycol dimeric copolymer. The phase transition behavior of the copolymer solution was observed using the inverted tube method and viscosity measurement method. It was found that the copolymer solution exhibits good fluidity at low temperatures and gels near body temperature. This technique is straightforward to implement, but its drawback is its inability to ascertain the phase transition temperature of thermally responsive polymers precisely. 

When choosing a testing method, certain factors should be considered, including the sample’s characteristics, the test’s purpose, the availability of equipment, and budgetary constraints. Various ways can offer varying types of information, and sometimes, combining methods can achieve more precise outcomes.

In conclusion, accurately determining the phase transition temperature of thermo-responsive polymers leads to better comprehension and regulation of these materials’ exceptional temperature-responsive characteristics, establishing a solid basis for their advancement and application in various fields.

### 3.2. The Methods for Controlling the Phase Transition Temperature of Thermally Responsive Polymers

After mastering the testing methods for the phase transition temperature of thermo-responsive polymers, the next step will be to explore in depth the procedures for regulating their phase transition temperature, which is crucial for optimizing the performance of thermo-responsive polymers and expanding their applicability in various applications. There are multiple means to control the phase transition temperature of thermo-responsive polymers, and this paper predominantly features five methods. 

#### 3.2.1. Controlling the Molecular Weight of Polymers

For different thermo-responsive polymers, the molecular weight of the polymer may have a particular impact on its transition temperature. According to the Flory–Huggins theory, for UCST thermo-responsive polymers, as Mn increases, ΔS_com_ increases, and the phase transition temperature rises accordingly [30]. On the contrary, for LCST thermo-responsive polymers, the larger the Mn, the lower the transition temperature. Ganorkar et al. [57] synthesized a copolymer with pH/temperature sensitivity, which can be used for drug loading and insulin release. The insulin release rate is controlled by controlling the copolymer’s molecular weight. The results show that the higher the molecular weight of the copolymer, the lower the phase transition temperature and the faster the insulin release rate.

#### 3.2.2. Controlling the Concentration of Polymers

The phase transition temperature of thermo-responsive polymers is also strongly influenced by the concentration of the polymer. When the polymer concentration is low, the distance between the polymer chains is too large to form a polymer network. The number of polymer aggregates increases when the polymer concentration is high enough. Therefore, polymers containing diverse chain segments interact, creating a polymer network that increases viscosity. Antunes et al. [13] found that when the polymer concentration is as low as 2 wt%, high viscosity values were not detected by increasing the temperature. When the attention of the polymer is higher than 5 wt%, the viscosity of the solution rises sharply. 

#### 3.2.3. Controlling the Degree of Polymer Crosslinking 

Changes in the degree of crosslinking in thermo-responsive polymer gel networks can regulate the behavior of gel swelling or shrinking at particular temperatures. Suzuki et al. [58] conducted tests measuring the transition temperature changes of PNIPAM gels with different cross-linking levels. Their findings reveal that as the degree of crosslinking decreases, the LCST of PNIPAM gels increases.

#### 3.2.4. Introduction of Copolymerization Monomers

By copolymerizing with hydrophilic or hydrophobic monomers and adjusting the polymer composition, the temperature sensitivity of the polymer can be significantly altered [59]. Seuring et al. [60] raised the phase transition temperature of NAGA by copolymerizing it with hydrophobic monomers such as Butyl Acrylate (BA) or Styrene (St) for diverse applications. 

Xue et al. [61] prepared copolymers of isopropyl acrylamide and different hydrophobic monomers by free radical polymerization and studied the phase transition temperature of these thermo-responsive polymer gels. The results showed that the stronger the hydrophobicity of the hydrophobic monomer, the lower the phase transition temperature of the copolymer. 

#### 3.2.5. Introduction of Additives

**Figure 5:**
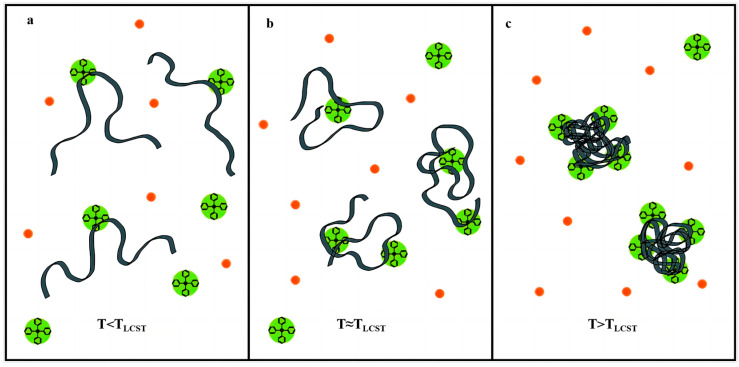
Interaction of tetrapheyl anions and neutral PNIPAM chains at different temperatures: (**a**) below the TLCS, Ph4B− ions feel an attraction for the hydrophobic moieties of the hydrated PNIPAM chains; (**b**) around the TLCS, the PNIPAM chains begin to dehydrate resulting in a more hydrophobic environment and the tetraphenyl anions increase their interaction with the PNIPAM interface; and (**c**) at T > TLCS, the great adsorption of the anions on PNIPAM chains limits the aggregation of the polymer and produces the formation of monodisperse nanoparticles with electrostatic stability [65]. (green is tetrapheyl anions, and red is salt ions).

Adding additives is an effective method to change the transition temperature of thermo-responsive polymers [62]. Zeng et al. [63] used Polyethylene Oxide-Polypropylene ether (P407 and P188), gum, and NaCl to prepare a thermo-responsive gel for sublingual administration of salbutamol. They studied the effects of different additives on the temperature sensitivity of the gel. They found that P407 lowered the gelation temperature, P188, and xanthan gum increased the gelation temperature, and NaCl decreased the gelation temperature. Umapathi et al. [64] studied the effects of different types of anions and cations in additives on the phase transition temperature of PNIPAM gel polymers, and the results showed that the anions in the additives had a significant effect on the LCST. Leonor et al. [65] studied the Interaction of tetrapheyl anions and neutral PNIPAM chains at different temperatures. The interaction of the tetraphenyl anions with the PNIPAM chains intensifies, ultimately promoting the formation of monodisperse nanoparticles with electrostatic stability at temperatures above the critical solution temperature. The shape of these particles constrains the hydrophobic interactions of the collapsing polymer chains, thereby reducing the LCST of PNIPAM (Figure 5).

The thorough testing and adjustment of the phase transition temperature of thermo-responsive polymers has resulted in a more complete understanding of the temperature-responsive properties of these materials. This provides a solid groundwork for further research into their potential and applicability in diverse practical contexts.

## 4. Application of Thermo-Responsive Polymers in Oil and Gas Extraction 

**Figure 6:**
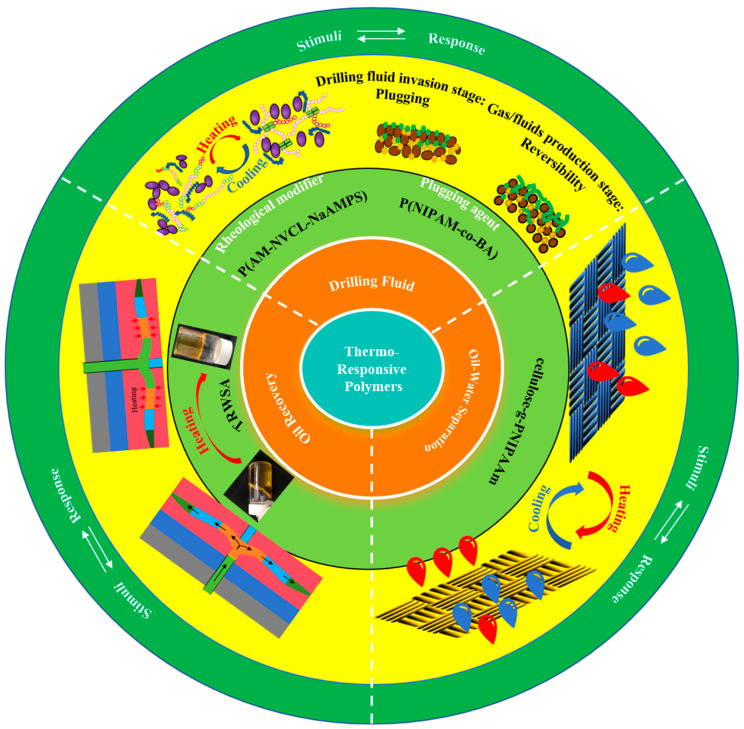
Application of thermo-responsive polymers in oil and gas extraction.

Oilfield chemicals are crucial for drilling, well completion, profile control, water plugging, and oil–water separation and are indispensable in oil and gas extraction. As oil and gas extraction moves towards increasingly complex and variable reservoir environments, the demand for high-quality oilfield chemicals continues to rise. Researchers have drawn on previous successes and advancements with thermo-responsive polymers in other fields to develop intelligent materials for oil and gas extraction [66,67,68,69,70,71,72], as shown in Figure 6. These materials display the advantageous properties of thermo-responsive polymers, resulting in high efficiency and environmental friendliness.

### 4.1. Application in Drilling Fluids

During drilling, drilling fluids perform essential functions such as cooling and lubricating tools, transporting drill cuttings, suspending solid-phase weighted materials, balancing formation pore pressures, and stabilizing well walls [73]. However, as the well depth increases, the wellbore temperature gradually rises, increasing the demand for higher-performing drilling fluids. Incorporating thermo-responsive polymers into drilling fluids aims to enhance their performance in various temperature and pressure scenarios, thereby addressing the complex obstacles encountered during deep well drilling [74].

#### 4.1.1. Application in Water-Based Drilling Fluid

Shale gas formations have a pore structure at the micro-nanometer scale, indicating that only particles of this scale can provide effective plugging and maintain well stability. Nanomaterials have been the pivotal research focus for using drilling fluid plugging agents in recent years. The drilling fluid’s ability to plug effectively is hindered by the formation of pore throat information, which depends on the proper sizing of the leakage channel and suitable matching of the bridging plugging material. Plugging failures can occur quickly if the plugging material particle size is unevenly matched [75,76]. Flexible plugging agents are greatly affected by formation temperature, lack high-temperature resistance, and have poor compatibility with drilling fluids [77]. Therefore, temperature-responsive microgel sealing agents have been applied in water-based shale drilling fluids capable of solidifying through a temperature-based stimulus response and possessing thermal reversibility.

**Figure 7:**
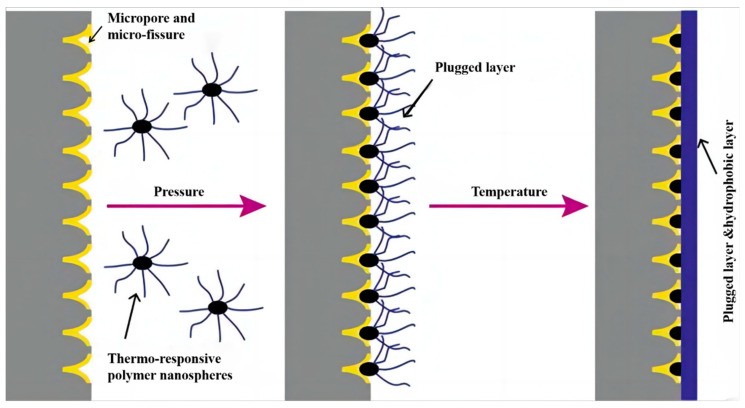
Schematic diagram of physical blockage and chemical inhibition of SD-SEAL [78].

Wang et al. [78] prepared a temperature-responsive microgel SD-SEAL, as shown in Figure 7. And when the temperature is higher than the LCST value, the SD-SEAL plays the dual role of physical plugging and chemical inhibition, which significantly reduces the permeability of the shale, slows down the pressure transfer, and improves the stability of the shale.

Bai et al. [79] developed a thermo-responsive gel plugging agent via reversed-phase suspension polymerization. This agent can significantly minimize the filtration loss of drilling fluids, enhance the tightness and hydrophilicity of filter cake, suppress the hydration expansion and dispersion of bentonite, and bolster the stability of shale well walls. 

Liu et al. [80] synthesized a thermo-responsive temporary plugging material in response to secondary damage to the oil formation caused by conventional displacement technology. The temperature-responsive quick plugging agent is appropriate for occurrences in the 70–90 °C temperature range. It possesses a high temporary plugging strength of 5–40 kPa and can be degraded in 1–15 days. The apparent viscosity after degradation is below 100 mPa-s, and the recovery rate of the simulated core permeability is more than 95%.

Zhao et al. [51] synthesized a thermo-responsive gel plugging material with a sol–gel–sol effect. The material’s main advantage is that it achieves blocking and unblocking through a change in the formation temperature without crosslinking or gel-breaking agents.

In deep-sea areas, the temperature is generally around 4 °C. The extremely low temperature dramatically affects the rheological properties of the drilling fluid system [81]. The viscosity of the drilling fluid increases sharply. The rheology is challenging to control, and the pipe column’s circulation resistance increases, dramatically affecting safe drilling in deep-sea environments with narrow density windows. At the same time, it dramatically impacts the suspension and sand-carrying performance of the drilling fluid. Therefore, controlling the low-temperature rheology of drilling fluids under deep-water conditions is a critical problem to solve in developing deep-water drilling fluids [82,83,84]. Thermo-responsive polymers can be used as rheology modifiers to be added to drilling fluids to achieve the constant rheology characteristics of deep-water drilling fluids due to significant fluid mechanics-based volume and molecular structure changes in the response process.

Hourdet et al. [85] prepared a thermo-responsive polymer (PAA-g-PEO), and the viscosity of the PAA-g-PEO solution varied with temperature, with significant temperature thickening. This discovery led to the application of thermo-responsive polymers in drilling fluids, enabling researchers and scholars to regulate the rheological properties of such fluids.

Ding et al. [86] synthesized a thermo-responsive copolymer with a wide temperature range, PNVDE. PNVDE can effectively control the rheological properties of deep-water water-based drilling fluids in the field of 4~60 °C, reducing the variation in viscosity and shear stress by about 20%. Additionally, PNVDE exhibits high temperature and salt resistance, allowing it to withstand fluctuations in temperature within drilling fluids.

Xie et al. [87] synthesized a thermo-responsive polymer, PANA. They adjusted the LCST of PANA by controlling the proportion of hydrophilic monomers, and the rheological properties of drilling fluids were more stable with the addition of PANA. The apparent viscosity changes, yield value, and low shear rate viscosity were less than 15% in the 4–75 °C temperature range. In comparison, the parameter changes in drilling fluids without adding PANA were close to or more than 30%.

Temperature-responsive copolymers as rheology modifiers are key treatment agents for achieving constant rheological properties in water-based drilling fluids and contain side chains with a critical solubility temperature (LCST), such as caprolactam. When the temperature is below the phase transition temperature, the thermo-responsive copolymer displays desirable water solubility in drilling fluids. However, suppose the temperature exceeds the phase transition temperature. In that case, the inter-conjugation of the hydrophobic side chains of the polymer enhances the entanglement, causing the viscosity of the solution to escalate. This results in the polymer exhibiting the characteristic of thermo-responsive thickening, which the viscosity of drilling fluids increases with the increase of temperature. 

#### 4.1.2. The Application in Oil-Based Drilling Fluids

In oilfield development, water-based drilling fluids are typically used for relatively easy-to-drill formations. However, oil-based drilling fluids are preferred for more challenging figures due to the various drilling incidents that can occur with water-based fluids. Compared to water-based fluids, the application of thermo-responsive polymers in oil-based drilling fluids is less common, primarily due to the unique chemical properties and application scenarios of oil-based fluids, which are often used in more complex or challenging drilling environments, such as under high-temperature and high-pressure (HTHP) conditions.

Wu et al. [88] developed a thermo-responsive expansive sealing material, TPA, for oil-based drilling fluid systems, addressing the technical challenges of wellbore instability and sound leakage encountered in drilling through the Ordovician formations in multiple ultra-deep wells in Shun Bei. This thermo-responsive material exhibits high temperature resistance and stability after oil absorption and expansion. At temperatures above 156 °C, it can expand more than 5.37 times, effectively addressing the size limitation issue for sealing materials in slim hole fracture reservoir sections and providing an effective technical solution for controlling drilling fluid loss in the fractured reservoir sections of ultra-deep wells.

Yang et al. [89] studied a new type of temperature-responsive polymer and analyzed its microstructure and morphology through methods such as Fourier transform infrared spectroscopy (FTIR), nuclear magnetic resonance (NMR), and transmission electron microscopy (TEM). They found that the polymer has a three-dimensional network structure and a particle size of 20–30 nm. This polymer can effectively regulate the viscosity of drilling fluids within a specific temperature range, serving as a rheological modifier for oil-based drilling fluids, thus enhancing their rheological properties.

Guo [90] synthesized an amphiphilic thermo-responsive nano-SiO_2_ hybrid material, PNM/SiO_2_-D, using a graft copolymerization method of thermo-responsive polymer monomers with modified nano-silica. This material exhibits more sensitive temperature-responsive behavior in emulsions with high water content. Upon reaching the phase transition temperature, it can effectively increase the viscosity of water-in-oil type oil-based drilling fluids, with the optimal addition amount being 1.5 wt%. The thickening effect is more pronounced under conditions of a higher water-to-oil ratio.

**Figure 8:**
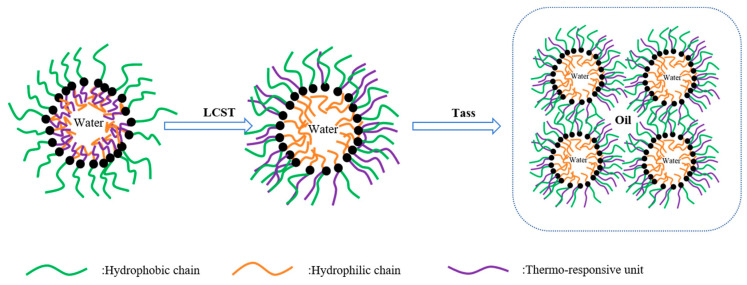
Schematic diagram of the viscosity enhancement mechanism of thermo-responsive SiO_2_ [90].

As illustrated in Figure 8, the thermo-responsive material PNM/SiO_2_-D is adsorbed on the interfacial film of the droplets in the emulsion, and when the temperature reaches LCST, the hydrophilic chain is transformed into the hydrophobic chain, which is transferred from the water phase to the oil phase. Upon reaching the Tass temperature, the molecular chains on the interfacial film of the droplets interlock, causing the viscosity of the emulsion to increase significantly.

While thermo-responsive polymers in oil-based drilling fluids are still in their early stages, they have substantial potential for addressing technical obstacles encountered during complex drilling operations, particularly those characterized by high temperatures and pressures. Nonetheless, their chemical compatibility with oil-based drilling fluids, cost-effectiveness ratio, and capacity to maintain performance stability in oil-based environments constrain their use. With the progress in materials research, particularly in the design of customized thermo-responsive polymers for oil-based drilling fluid systems, it is expected that these innovative materials will play an even more critical role in regulating the rheological properties of drilling fluids, stabilizing performance at high temperatures and high salinities, and controlling leakage. In the future, optimizing polymer design and improving their performance in oil-based drilling fluids will likely result in more significant application of thermo-responsive polymers in the oil and gas extraction industry. This has the potential to revolutionize the industry.

### 4.2. Applications in Oil Recovery

Thermo-responsive polymers, as intelligent materials, are transforming traditional oil recovery methods and strategies in the oil recovery process. Responding to temperature changes, they precisely control fluid flow in the formation. Their application in oil recovery garners increasing attention, particularly in water plugging, prevention of steam run-offs, and oil repulsion, which present significant potential.

#### 4.2.1. Profile Control and Water Plugging

High water content is prevalent in oil wells during oilfield development, particularly in the middle and later stages [91,92]. It is possible to improve the formation’s water absorption profile by injecting a system of water plugging and profile control agents. This will achieve the objective of increased oil production and decreased water content [93]. Commonly used profile control agents include gel, particulate, and precipitate types. However, conventional agents often need better adaptability for high-temperature, high-mineralization reservoirs, resulting in gel formation times that are too short to maintain good blocking performance [94,95,96]. The polymer gel system is cost-effective for enhancing fluid sweep efficiency, increasing oil recovery, and decreasing water production. However, conventional polymer microgels have limited application in complex and harsh reservoirs. Thermo-responsive polymer gel, an emerging intelligent polymer material, has become a significant area of research today. The gel molecules contain hydrophilic and hydrophobic groups in a specific ratio, and the temperature changes affect the interactions among these groups and hydrogen bonding, resulting in changes in the gel’s network structure. As a result, thermo-responsive polymer gels can act as profile control and water plugging agents in oilfield water applications. Thermo-responsive polymer gels possess the fundamental traits of traditional gels and facilitate selective plugging based on temperature field variations and reservoir distribution.

Zhao et al. [97] introduced a gel synthesized from novel associative polymers (AP) and crosslinking agents, suitable for profile control in high-temperature, high-salinity reservoirs like those in the Zhongyuan Oilfield. The gel maintains 75–85% of its original strength after six months at 95 °C and a mineralization level of 15×10^4^ mg/L. Field applications demonstrated its efficacy in sealing high-permeability layers, improving water injection profiles, enhancing the water absorption of low-permeability layers, increasing oil production, and reducing water cut, with an average increase of 203 T of oil per well and a cost–benefit ratio of 1:2.5.

Bai et al. [98] synthesized a temperature-responsive self-lubricating PAFB hydrogel for in-depth profile control. In displacement experiments at 120 °C, the plugging and recovery rates of PAFB reached 83.1% and 75.6%, respectively, demonstrating its excellent performance in deep profile control and enhancing oil recovery rates.

#### 4.2.2. Prevention and Control of Steam Channeling

Steam injection is commonly utilized to increase production yields during viscous crude oil production. Although effective in this regard, steam channeling problems can often plague oil reservoirs after multiple rounds of steam injection [99,100]. The formation of steam channels leads to reduced steam sweep volume, increased thermal energy loss, uneven reservoir exploitation, and heavy sand production, which significantly hinders the development of heavy oil reservoirs. Implementing measures to prevent or block steam channeling is crucial for improving recovery rates in heavy oil reservoirs [101]. Thermo-responsive polymer gels have advanced the development of plugging agents that effectively control steam channeling. These agents have yielded excellent results.

Altunina et al. [102] utilized thermo-responsive and polymer gels to regulate the distribution of injected thermal vapors during heavy oil’s thermal recovery, reduce water output, and increase oil mobility and displacement efficiency. As a result of field application, the gels increased steam coverage, lowered the water content of producing wells by 3–45%, and boosted oil production by 11–33%.

Yu et al. [103] have developed a new, cost-effective plugging agent system called SNM, capable of withstanding high temperatures in horizontal wells for heavy oil–steam throughput. The system has a viscosity of approximately 3 mPa·s at room temperature and excellent injection performance. It begins to solidify at 80 °C, eventually forming a solid block, and can withstand high temperatures up to 350 °C. The system has been successfully applied to several wells in the field.

Thermo-responsive reversible gel significantly affects the prevention and control of steam channeling, with a sound effect in field tests. The temperature-responsive gel can be applied directly into standard water injection wells, making it suitable for high-temperature and high-salt reservoirs. The gel has minimal loss during injection and changes from a solution to a gel state as the well temperature changes, resulting in efficient plugging of steam channels. When the wellbore temperature drops below its phase transition temperature, the thermo-responsive polymer gel can change into a solution, allowing oil and gas production to continue. However, the thermo-responsive gel transformation temperature is difficult to adjust, and the applicable interval is small, so it is difficult to popularize the thermo-responsive gel more widely.

### 4.3. Application in Oil–Water Separation

Substantial water injection into the formation is necessary for crude oil extraction. Following enhanced oil recovery measures such as polymer flooding and alkaline-surfactant-polymer (ASP) flooding, the fluids produced from oil fields tend to form W/O and O/W emulsions. These emulsions pose significant challenges for crude oil transportation, processing, and environmental protection. Therefore, effective oil–water separation of produced fluids is crucial for reducing transportation costs, preventing equipment corrosion, protecting the environment, improving crude oil quality, and ensuring the efficiency and safety of refinery operations. Stimuli-responsive emulsions, formed by intelligent, responsive materials that can emulsify or demulsify under external stimuli, facilitate the transport and recovery of heavy oil [104]. Temperature response is the most readily achievable, and based on this characteristic, researchers have developed a series of controllable wettability intelligent oil–water separation materials using temperature-responsive polymers.

**Figure 9:**
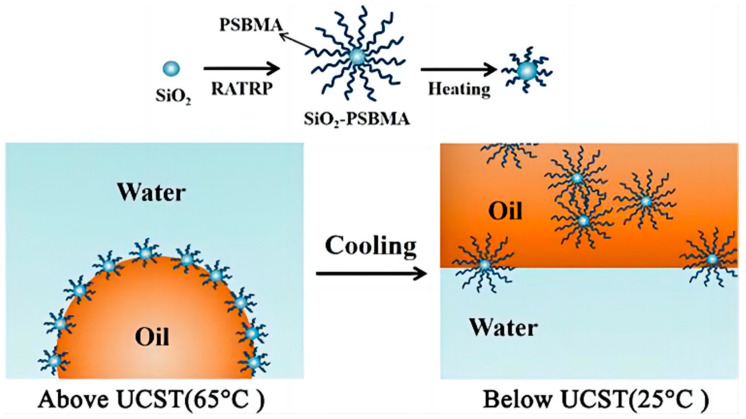
Response mechanism of thermo-responsive emulsified viscosity reducers [105].

Wang et al. [105] utilized reversible addition-fragmentation chain transfer polymerization (RATRP) to synthesize six groups of UCST temperature-responsive emulsifiers SiO_2_-PSBMA to reduce the viscosity of produced fluids (Figure 9). These emulsifiers’ upper critical solution temperatures ranged between 40 and 50 °C and could demulsify at room temperature. These temperature-responsive emulsifiers exhibited excellent emulsification effects, with the viscosity reduction rate (VRR) increasing and decreasing as particle size increased.

In experimental processes, the atom transfer radical polymerization (ATRP) method demands stringent reaction conditions and involves complex operations, hindering its widespread application. Zhang et al. [106] used a simple hydrothermal method to coat a layer of isopropyl acrylamide polymer on a nylon microfiltration membrane, producing a nylon membrane with reversible wettability. When the temperature is below the lower critical solution temperature (LCST, approximately 25 °C), the material exhibits hydrophilic and superoleophobic properties, suitable for separating various O/W emulsions. At temperatures above the LCST (about 45 °C), it exhibits hydrophobic and super oleophilic properties, effectively separating stable W/O emulsions. The separation efficiency of this membrane for various emulsions exceeds 97.8%, with excellent cyclic stability and high efficiency in separating oil–water emulsions.

Temperature-responsive materials for oil–water separation offer advantages such as ease of operation, rapid response, and no secondary pollution. The applicable temperature range of thermo-responsive polymers for oil–water separation is relatively narrow. Future research and development of new thermo-responsive polymers could enable effective operation over a broader temperature range, meeting the needs of different environmental and application scenarios. Thermo-responsive polymers have substantial potential for development and application in oil–water separation.

This section presents a systematic introduction to how thermo-responsive polymers are utilized in oil and gas extraction. However, certain thermo-responsive polymer materials need more sensitivity, challenges in controlling the phase transition temperature, and low resilience to temperature and salt. These concerns provide a direction for better future research in developing thermo-responsive polymers.

## 5. Conclusions

As oil and gas extraction moves to more complex reservoirs, the performance requirements for drilling fluids continue to rise. In this context, thermo-responsive polymers in oil-based drilling fluids exhibit significant potential, particularly in enhancing drilling efficiency, optimizing the stability of healthy walls, and improving the quality of mud cakes. Thermo-responsive polymers can act as intelligent plugging agents in oil-based drilling fluids, effectively sealing minor, unstable fractures in the healthy wall and maintaining stability. The polymers possess thermal sensitivity, undergoing a phase transformation upon reaching a specific temperature and transitioning to adapt to intricate formation conditions by impeding any blockage. Additionally, these substances modify the rheological properties of the drilling fluid while enduring high-temperature and high-pressure environments, which reduces filtration and optimizes drilling speed. With advancements in materials science, new thermo-responsive polymers are anticipated to be created, bringing innovative changes to oil-based drilling fluids due to their distinct temperature-responsive properties. 

Future research will aim to improve the stability and performance of thermo-responsive polymers in extreme oil-based drilling fluid environments, particularly at high temperatures, salinity, and pressure levels. A key focus will also be optimizing cost-effectiveness to ensure the practicality and economic feasibility of these high-performance materials. By studying thermo-responsive polymers that exhibit multiple functionalities, including resistance to high temperatures, salt, and mechanical stress, we can expect the emergence of efficient and multifunctional drilling fluid additives.

Incorporating thermo-responsive polymers in oil-based drilling fluids is anticipated to drive technological advancements in oil and gas field development, leading to a more efficient, safe, and eco-friendly process for extracting oil and gas. With the implementation of advanced synthetic technologies and a deeper comprehension of material properties, the distinctive function of thermo-responsive polymers within oil-based drilling fluids is poised to bolster the expansion of the oil and gas industry towards a more environmentally conscious and sustainable trajectory.

## Data Availability

The data presented in this study are openly available in article.
